# New products

**DOI:** 10.1155/S1463924698000133

**Published:** 1998


Journal of Automatic Chemistry, Vol. 20, No. 3 (May–June 1998) pp. 95–97

### New products

### On-line mercury analysis for process applications

P S Analytical, recognized as world leading manufacturers of atomic fluorescence systems for the determination of mercury and the hydride forming elements have launched their new generation on-line analyser. The 10.223 on-line mercury analyser is for the continous determination of mercury in flowing liquid streams. Designed for unattended operation, the 10.223 offers unsurpassed sensitivity and linearity and can be configured for the analysis of most sample types for multiple sample streams.

Each installation offers a guaranteed solution to the customer's problem, even for the most complicated of sample matrices. The system can be customized in terms of sampling, alarms, report generation and integration into the plant control room. Discrete analytical measurements are made in fixed or variable sequence, rather than measuring a level continuously. This overcomes many interference problems associated with very complex matrices.

*Further information from P S Analytical Ltd, Arthur House, Crayfields Industrial Estate, Main Road, Orpington, Kent BR5 3HP, UK. Tel.: 01689 891211; fax: 01689 896009; email: psa@psanalytical.demon.co.uk.*

### ICP-MS

Micromass have begun shipping their Platform ICP and *iso*-PlasmaTrace systems, in addition to the already successful PlasmaTrace 2.

The *Platform ICP* was the first instrument to use the technique of ICP-HEX-MS to eliminate argon-based interferences and it has been awarded the PittCon Editors' Gold Award for Best New Product. Initial development by Alan Gray in the early 1980s showed the ICP-MS technique to be plagued by interferences on what, for many applications, are ironically the most important elements (potassium, calcium, chromium, manganese, iron, arsenic, and selenium). Such interferences resulted from the formation of molecular species in the plasma; combinations of matrix elements such as oxygen with argon. The presence of these interferences gave rise to an elevated background at the analyte mass which, at best, raised the achievable detection limits and, at worst, rendered the analyte isotope unusable.

Platform ICP incorporates HEX™ de-clustering. The procedure involves a hexapole ion lens, located behind the skimmer cone, being surrounded by a gas cell. By admitting small amounts of helium, the pressure over the length of the hexapole is increased, breaking up molecular species before they reach the mass analyser, while the hexapole lens reduces the energy spread of the ions as they emerge from the skimmer. Detection limits for most elements are in the ppt range, including previously difficult elements such as K, Ca, Fe, Se and As.

Platform ICP uses a new type of detector for ICP-MS. Micromass' Dynolite™ detector technology is a wide dynamic range photomultiplier detector, which gives eight orders of magnitude without changing detection mode thereby eliminating cross calibration and reducing analysis times. The detector has an estimated lifetime of 10 years, compared to typically one–two years for conventional ICP-MS detectors.

*PlasmaTrace 2* uses a high resolution reverse geometry to optimize transmission and sensitivity at very high resolution levels. However, routine, accurate determinations may still be carried out from ppm to ultratrace (ppq) levels for most elements. Inclusion of the PlasmaDrive command station ensures that the operator has fingertip controls, a facility which allows full access to instrument parameter adjustment and monitoring. Other advanced features include the Sprint rapid scan facility, dual detector system and the unique Auto Res™, which automatically adjusts the working resolution of PlasmaTrace 2 during analysis.

*iso-PlasmaTrace* is a multicollector ICP-MS system, which uses an array of detectors placed in defined positions, enabling simultaneous determination of all analyte isotopes. In addition to eliminating argon interferences, the HEX™ system dramatically reduces the energy spread of ions entering the analyser. This enables iso-PlasmaTrace to use a single focusing sector analyser as opposed to the more complex and more expensive double focusing analysers. iso-PlasmaTrace also ensures that all of the signal for each isotope is counted because the analyser does not have to scan between masses, resulting in increased sensitivity and lower detection limits for measuring ratios.

*Further information from Micromass UK Ltd, Floats Road, Wythenshawe, Manchester M23 9LZ, UK. Tel.: 0161 945 4170; fax: 0161 998 8915; email: andrew.eaton@micromass.co.uk.*

### Spectral search package

Galactic Industries launched its new 32-bit spectral searching application Spectral ID™ at the 1998 Pittsburgh Conference in New Orleans. Spectral ID is the only search package to support all the popular commercial libraries including Aldrich, Nicolet-Aldrich, Sadtler, Sprouse, and Fiveash Data Management. Users can search multiple libraries of different ranges and resolutions and even save favourite sets of libraries for future searches. Custom libraries can be easily defined and made up of virtually any optical data type at any spectral range or resolution.

0142-0453/98 $12·00 © 1998 Taylor & Francis Ltd

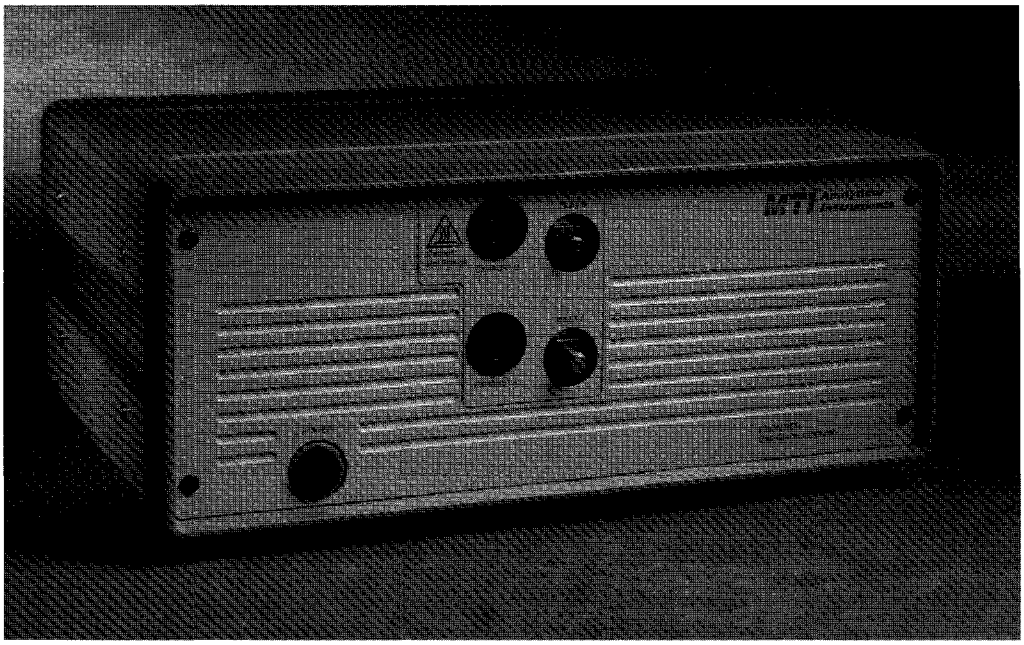

**MTI's Frontier high-speed gas chromatograph combines speed, sensitivity and modularity**

MTI Analytical Instruments® ■ 41762 Christy Street ■ Fremont, CA 94538 ■ (510) 490-0900 ■ Fax (510) 490-0904 ■ www.mtigc.com

*Frontier is a high speed gas chromatograph system with a new picogram sensitive Flame Ionization Detector (FID). The Frontier is chassis and tray design, allows for injector, column, detector and electrometer replacement in the field in less than 5 mins. A silicon micromachined injector has been designed specifically to meet the demands of low detection limit work, with sub-100 ppb detection capabilities. The Frontier takes advantage of many recent advancements in inert material production. One example is surface deactivation of the entire sample pathway by the deposition of an inert layer called SilcoSteel, developed by Restek Corporation.*

*Details from MTI Analytical Instruments, 41762 Christy Street, Fremont, CA 94538, USA. email: http://www.mtigc.com.*

Spectral ID reaches speeds of over 4000 compounds per second on full spectrum searches, and virtually instantaneous peak and text searches. Even using the Region Mask, a feature that allows users to include or exclude an unlimited number of spectral regions from a search, Spectral ID searches just as fast as without a mask.

Spectral ID offers seven industry-standard full spectrum search algorithms, including the correlation and 1st derivative correlation, two methods of peak searching, and flexible text query methods. The full spectrum, peak, or text searching methods can be used alone or in combination to optimize search results.

*Details from Galactic, 395 Main Street, Salem, NH 03079-2464, USA. Tel.: 603 898 7600; fax: 603 898 6228; email: http://www.galactic.com.*

### Excalibur

The Millennium Excalibur is controlled directly by an RS232 interface which allows very flexible control of the operating conditions, including pump speed, etc. A custom-built moisture removal system with a cassette design allows significant improvement in the signal to noise ratio, which has already had the benefit from better pump flow control.

The Excalibur system also has an integrated flame sensor which insures against loss of sample or blank whenever the flame is not present. This instrument is simple and easy to use and access to the lamp housing is directly through the front cover, so it is easy to switch between one analyte and another. Detection levels achieved for a range of elements are:

| *Element* | *Limit of detection* |
|---|---|
| Se | 2ppt |
| As | 10ppt |
| Sb | 10ppt |
| Te | 10ppt* |
| Bi | 10ppt* |

*With optimized multi-reflectance filter.

*Further details from PS Analytical (as before).*

### Chromatography data systems

Justice Laboratory Software has introduced the Chrom Perfect NT chromatography data system, a 32-bit application designed specifically for Windows NT 4.0 and Windows 95. The Chrom Perfect NT can be supplied configured for laboratories that have one to hundreds of LCs and GCs. Ease-of-use is enhanced by extensive use of standard Windows NT methods, such as long-file names, drag-and-drop, tool help, and enhanced metafiles. For the administration of larger systems there is centralized password administration. Chrom Perfect NT retains many of the time-tested integration, reporting, calibration, and graphical interface features of earlier 16-bit versions of Chrom Perfect, while providing the ability to read data files collected by earlier versions. Data can be acquired from 24-bit A/D boards, HP 6890/5890 GCs (including full instrument control), and PE-Nelson interfaces to provide a common environment for any kind of chromatograph from any manufacturer.

*For more information contact James Ressell, J. L. S. House, 27 Low Road, Auchtermuchty, Fife KY14 7BB, UK. Tel.: 01337 828494; fax: 01337 828404; email: JLabUK@AOL.com.*

### Mercury determinations in natural gas and condensates

The problems associated with mercury contamination in natural gas and condensate samples are well recognized, but few acceptable methods have been validated for the reliable determination of these levels. Since the introduction of the Sir Galahad instrument in 1991, P S Analytical has worked closely with a number of petrochemical companies, in particular with Amoco, to develop reliable sampling protocols and accurate determinations. Many of these companies have now appointed PSA as the sole provider of analytical measurement systems.

Recently, PSA has introduced two simple sampling systems for use in the natural gas markets. These provide both offline and online sampling systems linked to the Sir Galahad instrument. Further work with the University of Plymouth, in association with Petronas Malaysia, has shown that the Sir Galahad instrument can also be readily adapted to measure the local levels of mercury in condensates. A wide range of samples has been tested using a simple technique involving no sample preparation. Accurate and reliable data has been obtained with detection levels below 50ppt in the condensates.

*For further information contact PS Analytical (as before).*

### High accuracy measurement of dispersions

The Turbiscan MA 1000 gathers information on the structure of dispersions, including their homogeneity and stability. The instrument produces data quickly using a non-contact procedure. It operates by vertically scanning the sample and measuring the transmitted and back-scattered light. It analyses the destabilization mechanisms in order to detect variations in particle size (coalescence, flocculation) and particle migration (creaming, sedimentation). Measurements can be made in static or kinetic mode. In kinetic mode, the tube of dispersion is placed in the Turbiscan MA 1000 and scanned several times over a single period of time to obtain a diagram of nascent phenomena. This is efficient if the dispersion is destabilized fairly quickly. For slower phenomena, the tube is placed in the instrument, scanned once and then removed. This process is repeated at regular intervals and the aggregation of the results over time produces a graph of the destabilization phenomena. The instrument can detect nascent segregation between four and 50 times quicker than with the naked eye. Dilution is not required over a concentration range from 0 to 70% w/w.

Applications include cosmetics, pharmaceuticals, agrofood, pigments, paints, bitumens, surfactants, etc.

*Details from Mme Isabelle Cayre, FORMULACTION, Thégone, 10 avenue de l'Europe, 31525 Ramonville-Saint-Ange, France. Tel.: 00 33 5 61 28 56 52; fax: 00 33 5 61 28 56 77.*

### 2nd Euroconference on Environmental Analytical Chemistry: Environmental Analytical Chemistry for the Protection of Sensitive Ecosystems

The Second Euroconference on Environmental Analytical Chemistry will discuss the role of analytical measurement processes in providing reliable chemical information with a view to taking effective social, economic and/or legal action in order to protect sensitive ecosystems. Forty fellowships covering accommodation with full board will be made available for young researchers from EU member and associated states. Twenty researchers from less favoured regions are eligible for a contribution towards the costs of travel of 40 000 ptas.

*Further information from Dra. Soledad Rubio, Department of Analytical Chemistry, University of Córdoba, E-14004 Córdoba, Spain. Tel.: 34 57 211066; fax: 34-57-218606; email: qa1rubrs@lucano.uco.es.*

